# MIHARI project, a preceding study of MID-NET, adverse event detection database of Ministry Health of Japan—Validation study of the signal detection of adverse events of drugs using export data from EMR and medical claim data

**DOI:** 10.1371/journal.pone.0255863

**Published:** 2021-09-08

**Authors:** Hiroshi Watanabe, Kiyoteru Takenouchi, Michio Kimura

**Affiliations:** 1 National Center for Geriatrics and Gerontology, Obu, Japan; 2 Consortium for SS-MIX Dissemination and Promotion, Kawasaki, Japan; 3 Hamamatsu University School of Medicine, Shizuoka, Japan; National Chiao Tung University College of Biological Science and Technology, TAIWAN

## Abstract

We studied the effectiveness of the direct data collection from electronic medical records (EMR) when it is used for monitoring adverse drug events and also detection of already known adverse events. In this study, medical claim data and SS-MIX2 standardized storage data were used to identify four diseases (diabetes, dyslipidemia, hyperthyroidism, and acute renal failure) and the validity of the outcome definitions was evaluated by calculating positive predictive values (PPV). The maximum positive predictive value (PPV) for diabetes based on medical claim data was 40.7% and that based on prescription data from SS-MIX2 Standardized Storage was 44.7%. The PPV for dyslipidemia was 50% or higher under either of the conditions. The PPV for hyperthyroidism based on disease name data alone was 20–30%, but exceeded 60% when prescription data was included in the evaluation. Acute renal failure was evaluated using information from medical records in addition to the data. The PPV for acute renal failure based on the data of disease names and laboratory examination results was slightly higher at 53.7% and increased to 80–90% when patients who previously had a high serum creatinine (Cre) level were excluded. When defining a disease, it is important to include the condition specific to the disease; furthermore, it is very useful if laboratory examination results are also included. Therefore, the inclusion of laboratory examination results in the definitions, as in the present study, was considered very useful for the analysis of multi-center SS-MIX2 standardized storage data.

## Introduction

A number of studies have attempted to utilize secondary data directly from EMR for clinical research, etc. A potential solution to this problem was first demonstrated by the Adverse Spontaneous Triggered Event Reporting (ASTER) Project [1]. ASTER enables adverse event reporting through current electronic health records (EHR) systems. When a doctor discontinues prescription of a drug for a patient, s/he is prompted with a series of short questions to determine if this is due to an adverse event from the drug. ASTER offers the opportunity to follow event details that are not recorded in an EHR, as it is not regulatory information. After about 20 minutes, a MedWatch report, derived from the EHR, is delivered to the Food and Drug Administration (FDA) to report on the adverse event. When ASTER was tested previously, 20% of these reports were deemed as "serious," 100% had height/weight and lab data, and 91% of participating physicians had not submitted any adverse drug event (ADE) reports the prior year. ASTER’s ease of use, and the opportunity to report adverse events at the point of care within 60 seconds (vs. 34 minutes for a fax report), makes this innovative approach an essential step in enabling safer, more effective drugs.

A recent publication of Nordo et al. [2] demonstrated that the use of the Integrating the Healthcare Enterprise® (IHE) Retrieve Form for Data Capture (RFD) Profile as a part of the Epic EHR research model along with the Research Electronic Data Capture (REDCap) electronic data capture (EDC) system and middleware (RADaptor) developed by the Duke University Office of Research Informatics produced significant time and resource savings and improved quality. Specifically, this eSource pilot for a registry study produced a 37% time savings and required one less full-time employee while the error rate was reduced from 9% to 0. For the Duke study, data elements mapped with RADaptor included those contained within the EHR’s continuity of care document (CCD), a standard used to comply with initial U.S. Meaningful Use requirements. Data elements not in CCD could be entered anew into the electronic case report form (eCRF).

Both of the above studies were conducted at each single institution. A multi-center study, known as the Electronic Health Record for Clinical Research (EHR4CR) project, was conducted in Europe [3]. The collection of data for this project required substantial effort because the EMR data had not been standardized. In order to obtain high-quality EMR data at low cost from multiple institutions for the purpose of surveillance, it is essential to establish a system that can collect standardized and structured data from EMR. In Japan, output of medical claim data is obtained using receipt codes standardized by the computerized receipt processing system in almost all institutions.

**Fig 1:**
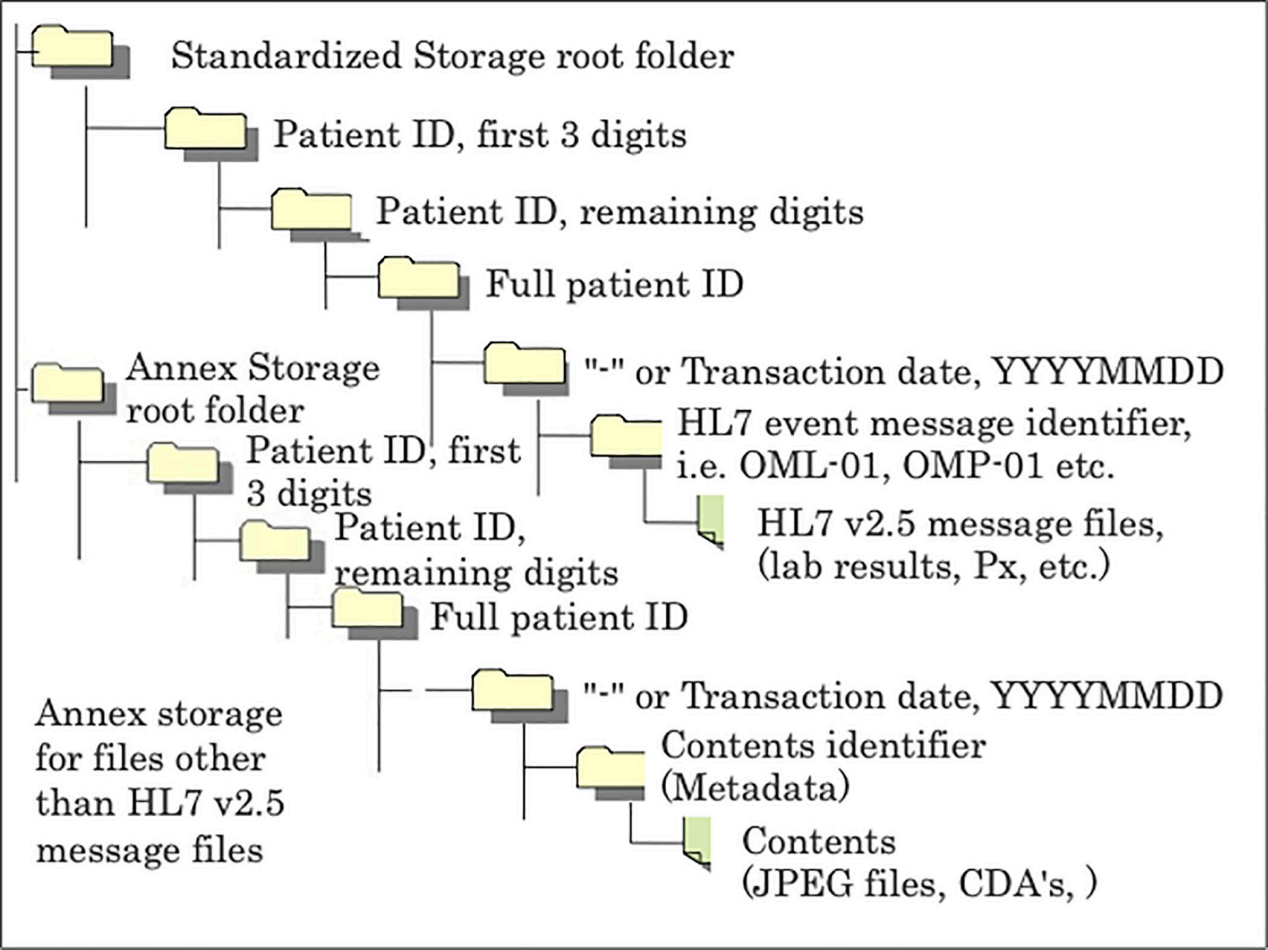
Description of the schematic structure of SS-MIX2 standardized storage and annex storage.

Standardized Structured Medical Information eXchange2 (SS-MIX2) Storages were used as the export data from EMR [4]. The SS-MIX project was promoted by Japan’s Ministry of Health, Labor and Welfare (MHLW) and was inherited from The Shizuoka Style EMR project in 2006FY [5]. According to investigations completed by MHLW in 2015FY [6], EMR systems were operating in 2,542 hospitals (34%) out of 7,426 hospitals in Japan and SS-MIX2 Standardized Storage was being implemented in 865 hospitals (34% of the hospitals with operational EMR systems). Confining these metrics to 710 hospitals with more than 400 beds, EMR systems were operating in 550 hospitals (78%) and SS-MIX2 Standardized Storage in 237 hospitals (43% of hospitals with EMR). SS-MIX2 Standardized Storage and Annex Storage is text-data-files storage that stores minimal medical information written by standard code, using multi-hierarchical folder structure (Fig 1: *Description of the schematic structure of SS-MIX2 Standardized Storage and Annex Storage*).

"SS-MIX2 Standardized Storage: explanation of the structure and guidelines for implementation Ver. 1.2" [7] was authorized as the standard specification of MHLW on 28 March 2016 [8]. Six hundred and thirty hospitals in Japan are storing patient demographics, diagnostic disease classification in ICD-10, prescription orders, and laboratory examination results in HL7 v2.5 format using SS-MIX2 Standardized Storage. Thus, the SS-MIX2 Specification is the de facto standard for the export from EMR in Japan. Hori et al. reported the detection of fluoroquinolone-induced tendon disorders by the secondary use of SS-MIX2 Standardized Storage at an institution [9].

A study on the early detection of drug-related adverse events using standardized patient data (i.e., medical claim data and the data from SS-MIX2 Standardized Storage) from multiple institutions was conducted by Japan’s Pharmaceuticals and Medical Devices Agency (PMDA). Here, we describe this Medical Information for Risk Assessment Initiative (MIHARI) project in detail. In this study, we investigated whether known adverse events could be detected from the data of several institutions that use SS-MIX2 standardized storage and compared the results with those from paper-based medical records and electronic data capture (EDC) systems. The database search engine D*D [10] was used in this study. It utilizes CACHE, which is a tree structure [11].

Based on its usefulness, the PMDA initiated a project named Medical Information Database Network (MID-NET) in 2018 for the detection of adverse events, in an attempt to substitute post-marketing surveillance [12–14]. In this paper, we describe the MIHARI project, in which we investigated whether known adverse events could be detected from data directly collected from the EMR of multiple institutions; this formed the basis of the ongoing MID-MET project.

## Materials and methods

To investigate whether known adverse events can be detected by database surveillance using a hospital information system, serving as a substitute for traditional large-scale surveys in which data from each hospital is individually collected by companies, etc., utilizing a large amount of resources.

**Fig 2:**
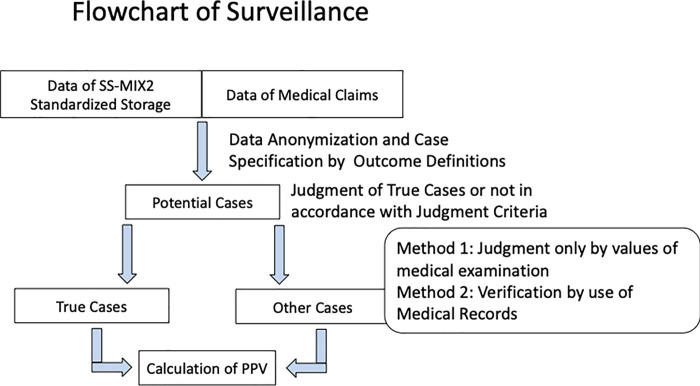
Methods for surveillance.

In this study, medical claim data and SS-MIX2 standardized storage data were used to identify four diseases (diabetes, dyslipidemia, hyperthyroidism, and acute renal failure) and the validity of the outcome definitions was evaluated by calculating positive predictive values (PPV) as indices of validity (Fig 2: *Methods for Surveillance)*.

Fig 2 shows the flowchart of surveillance that combination of medical claim data and SS-MIX2 standardized storage data are used for calculation of potential cases after anonymization.

In this study, we used two methods. Method 1 was used to identify diabetes, dyslipidemia, and hyperthyroidism. Method 2 was used to identify acute renal failure.

- Method 1: Evaluation of validity based only on laboratory examination results.

Subject outcomes: New onset of diabetes, dyslipidemia, and hyperthyroidism.

- Method 2: Evaluation of validity based on data, including information from medical records (medical records were reviewed by physicians with the support of clinical research coordinators [CRC]).

Subject outcome: New onset of acute renal failure.

We adopted two different evaluation methods because the validation study of Method 2, including data from medical records, would require a large amount of resources in terms of time and cost for reviewing; therefore, we also used a simple method (Method 1) based only on laboratory examination results for the evaluation of validity of the outcome definitions.

{Data sources}

SS-MIX2 standardized storage data were obtained from the hospitals listed below. Medical claim data and medical records were obtained from two hospitals among them.

- ・National University Corporation Kyushu University Hospital
- ・Social Welfare Organization Saiseikai Imperial Gift Foundation, Inc., Saiseikai Shizuoka General Hospital
- ・Shizuoka Prefectural Hospital Organization, Shizuoka General Hospital
- ・Numazu City Hospital
- ・National University Corporation Hamamatsu University Hospital
- ・Fukuroi City Hospital

{Period of data surveyed}

From April 1, 2007 to December 31, 2011

{Anonymization of data}

A second PMDA identification (ID) was assigned to each patient whose data were extracted. In Method 1, irreversible anonymization was performed, before providing the data to PMDA, by discarding the correspondence table between original hospital ID and second ID. In Method 2, despite the fact that irreversible anonymization was desirable because their medical records were utilized, the correspondence table between original hospital ID and second ID was kept under lock and key in each hospital so that the patient data was not identified by outsiders.

{Case identification by outcome definitions}

Codes were provided for each disease name, drug, and medical practice according to disease. Some of the codes are provided in Table 1 (*Examples of Code Lists)*. International Statistical Classification of Diseases and Related Health Problems-10 (ICD-10) codes were used for disease names, YJ codes were used for drugs, and the codes of the computerized receipt processing system were used for the related medical practice.

**Table 1:** Examples of code lists.

**Example of Code List of Disease Name**			
**ICD-10 Small Classification**		**Standard Disease Names**	**Codes for Computerized Processing of Medical Claims**
E10	Insulin Dependent Diabetes Mellitus (IDDM)	Type 1 diabetes mellitus	2500014
		Slowly progressive insulin-dependent diabetes mellitus	8844022
		Brittle diabetes mellitus	2500027
**Example of List of Drug Codes**			
	**YJ Code**	**Drug Form**	**Ingredient Name**
	**(Initial 7 digits are the same as those of National Health Insurance Drug List)**		
Antithyroid Drugs	2432001	Oral Drug	Thiamazole
	2432400	Injection Drug	Thiamazole
	2432002	Oral Drug	Propylthiouracil
Example of Code List of Medical Examination			
	**Codes for Computerized Processing of Medical Claims**	**Name of Medical Practices**	
For Medical Examination	2432001	Triglyceride	
	2432400	Total Cholesterol	
	2432002	HDL-Cholesterol	

Using these codes, automatically extracted groups (potential cases) were identified based on eight extraction conditions (outcome definitions) for Method 1 (Table 2: *Outcome Definition in Method 1*) and one extraction condition (outcome definition) for Method 2 (Table 3: *Outcome Definition in Method 2*).

**Table 2:** Outcome definition in Method 1.

Outcome Definition 1)	Data Source Used		Temporal relationship between items 2)	Index Month
	SS-MIX2	Medical Claim		
Definition 1	Y	Y	-	Month of initial definitive diagnosis
Disease Name Only				
Definition 2	Y	Y	-	Months of initial drug prescription
Drug Only				
Definition 3–1	Y	Y	The month of initial definitive diagnosis is the same as or that prior to the month of initial drug prescription. No interval limitation between the month of initial definitive diagnosis and that of initial drug prescription.	Months of initial drug prescription
Disease Name and Drug				
(No interval limitation)				
Definition 3–2	Y	Y	The month of initial definitive diagnosis is the same as or that prior to the month of initial drug prescription. The interval between the time of initial definitive diagnosis and the time of initial drug prescription was 1 month or shorter.	Months of initial drug prescription
Disease Name and Drug				
(Interval limitation)				
Definition 4		Y	The month of initial definitive diagnosis is the same as the month of medical practice.	Month of initial definitive diagnosis
Disease Name and Medical Practice				
Definition 5		Y	The month of initial definitive diagnosis was the same as the month of medical practice.	Months of initial drug prescription
Drug and Medical Practice				
Definition 6–1		Y	The month of initial definitive diagnosis is the same as or before the months of medical practice, and the month of medical practice is the same as or before the month of initial drug prescription. No interval limitation between the month of initial definitive diagnosis and the month of initial drug prescription.	Months of initial drug prescription
Disease Name, Medical Practice, and Drug				
(No interval limitation)				
Definition 6–2		Y	The month of initial definitive diagnosis is the same as or before the months of medical practice, and the month of medical practice is the same as or before the month of initial drug prescription. The interval between the time of initial definitive diagnosis and the time of initial drug prescription was 1 month or shorter.	Months of initial drug prescription
Disease Name, Medical Practice, and Drug				
(Interval limitation)				

1) Disease names, treatment drugs, and medical practice used for the search were shown in Appendix 1, 2, and 3.

2) Month of initial definitive diagnosis: The month in which the disease used for the definition was initially diagnosed during the data period.

3) Month of initial drug prescription: The month in which the drug used for the definition was initially prescribed during the data period.

**Table 3:** Outcome definition in Method 2.

Outcome Definition 1)	Used Data Source		Temporal relationship between items 2)
	SS-MIX2	Medical Claim	
Definition 1	Y		Acute elevation of serum Cre levels 3) within 1 month prior to and after the month of initial definitive diagnosis.
Disease Name and Rapid Increase in Serum Creatinine Values			

1) Disease names used for the search are shown in Appendix 1.

2) Month of initial definitive diagnosis: The month in which the disease used for the definition was initially diagnosed during the data period. Month of initial drug prescription: The month in which the drug used for the definition was initially prescribed during the data period.

3) The definition of acute elevation of serum Cre levels: The serum Cre level within 1 month before and after the index month increased by 0.3 mg/dL or up to 150% compared to the previous serum Cre level of the past 3 months.

In Method 1, the outcome definitions differed according to data source (medical claim data alone or the use of SS-MIX2 standardized storage data) and the combination of disease name, drug, and medical practice.

In Method 2, the outcome definition was based on the combination of disease name and laboratory examination data obtained only from SS-MIX2 standardized storage data.

{True case identification}

“True cases” were identified from the extracted potential cases.

Method 1 (diabetes, dyslipidemia, and hyperthyroidism).

The case identification method based on laboratory examination results was adopted, and the method was described with reference to the diagnostic guidelines and the opinions of clinicians (Table 4: *The case identification method*).

**Table 4:** The case identification method.

The case identification method for diabetes
1.Fasting blood glucose level is 126 mg/dL or higher or casual blood glucose level is 200 mg/dL or higher.
2.HbA1c is 6.1% or higher.
Identified as a true case if the above criteria were met twice or more during the observation period.
The case identification method for dyslipidemia
1.Blood LDL cholesterol level is 140 mg/dL or higher.
2.Blood HDL cholesterol level is less than 40 mg/dL.
3.Blood triglyceride level is 150 mg/dL or higher.
Identified as a true case if the above criteria were met at least once during the observation period.
The case identification method for hyperthyroidism
1.The free T4 level is 1.8 ng/dL or higher or the free T3 level is 4.0 ng/dL or higher.
2.Blood TSH level is 0.1 μU/mL or lower.
Identified as a true case if both criteria were met.

Method 2 (Acute renal failure)

True cases were identified based on medical records, with reference to laboratory examination results after consultation with clinicians.

The case identification method for acute renal failure

Cases of acute renal failure were identified by three nephrologists based on acute changes in serum creatinine (Cre) levels.

We decided that patients on long-term dialysis and those who had undergone renal transplantation should be excluded from the analysis. Therefore, patients were divided into subgroups according to previous serum Cre levels, according to laboratory tests in the past 3 months, in order to identify those who previously had high serum Cre levels.

The previous serum Cr levels were classified into three categories: ≦1.2 mg/dL, >1.2 mg/dL and ≦2.0 mg/dL, and >2.0 mg/dL.

Cases were classified as “true cases” or “other cases” according to the identification method for each disease. Subsequently, PPV and 95% confidence intervals (95% CI) were calculated as indices for the evaluation of the validity of the outcome definitions. This time, the Wald method was adopted to estimate the confidence interval [15]. The formula used for calculating PPV is as follows:

PPV% = number of patients classified as true cases/number of potential cases × 100.

In Method 2 (acute renal failure), true cases were identified from random samples, accounting for 30% of total potential cases. The following formula was used in this case for PPV calculation:

PPV% = number of patients classified as true cases/number of subjects × 100.

{Ethical background}

This trial survey uses the secondary data of electronic medical record stored in the hospital information system in daily medical care. The trial survey was conducted as follows: “Ethical Guidelines for Epidemiological Research June 17, 2002 (Revised December 28, 2004) (Revised June 29, 2005) (Revised August 16, 2007)) (Partial revision on December 1, 2008)”.

The implementation of this trial was consulted and approved by the Ethics Review Committee of the International University of Health and Welfare (approval number: 11–169).

## Results

With respect to the outcome definition for newly developed diabetes, the number of cases and PPV based on medical claim data and SS-MIX2 standardized storage data are shown in Table 5 (*Number of cases and PPV according to the outcome definition for diabetes)*. With respect to the outcome definition for diabetes, none of the PPVs reached 50% under any condition. The maximum PPV for “Definition 3–2 Disease Name and Drug (No interval limitation)” using the SS-MIX2 standardized storage data was 44.7%, whereas the maximum PPV for “Definition 6–2 Disease Name, Medical Treatment, and Drug (No interval limitation)” using medical claim data was 40.7%. The PPVs for diabetes were higher among the results obtained from SS-MIX2 standardized storage data than those obtained from medical claim data in any of the definitions.

**Table 5:** Number of cases and PPV according to the outcome definition for diabetes.

Outcome Definition	Potential Case	True Case	Other Case	PPV
Data Source	A	B	C	B/A % (95%CI)
Definition 1				
SS-MIX2 Standardized Storage Data	22086	4646	17440	21.0% (20.5–21.6)
Medical Claim Data	10647	1902	8745	17.9% (17.1–18.6)
Definition 2				
SS-MIX2 Standardized Storage Data	11947	4538	7409	38.0% (37.1–38.9)
Medical Claim Data	6854	2043	4811	29.8% (28.7–30.9)
Definition 3–1				
SS-MIX2 Standardized Storage Data	8092	3671	4421	45.4% (44.3–46.5)
Medical Claim Data	4341	1696	2645	39.1% (37.6–40.5)
Definition 3–2				
SS-MIX2 Standardized Storage Data	5212	2329	2883	44.7% (43.3–46.0)
Medical Claim Data	2909	1149	1760	39.5% (37.7–41.3)
Definition 4 Medical Claim Data	9740	1831	7909	18.8% (18.0–19.6)
Definition 5 Medical Claim Data	6202	1968	4234	31.7% (30.6–32.9)
Definition 6–1 Medical Claim Data	4215	1680	2535	39.9% (38.4–41.3)
Definition 6–2 Medical Claim Data	2785	1133	1652	40.7% (38.9–42.5)

Considering the outcome definition for newly developed dyslipidemia, the number of cases and PPV calculated using medical claim data and SS-MIX2 standardized storage data are shown in Table 6 (*Number of cases and PPV according to the outcome definition for dyslipidemia*). The PPV for dyslipidemia was 50% or higher under all of the conditions.

**Table 6:** Number of cases and PPV according to the outcome definition for dyslipidemia.

Outcome Definition	Potential Case	True Case	Other Case	PPV
Data Source	A	B	C	B/A % (95%CI)
Definition 1				
SS-MIX2 Standardized Storage Data	17721	9588	8133	54.1% (53.4–54.8)
Medical Claim Data	8867	5276	3591	59.5% (58.5–60.5)
Definition 2				
SS-MIX2 Standardized Storage Data	14185	8762	5423	61.8% (61.0–62.6)
Medical Claim Data	7596	4795	2801	63.1% (62.0–64.2)
Definition 3–1				
SS-MIX2 Standardized Storage Data	10586	6752	3834	63.8% (62.9–64.7)
Medical Claim Data	6218	3988	2230	64.1% (62.9–65.3)
Definition 3–2				
SS-MIX2 Standardized Storage Data	8938	5581	3357	62.4% (61.4–63.4)
Medical Claim Data	5511	3450	2061	62.6% (61.3–63.9)
Definition 4 Medical Claim Data	6756	4635	2121	68.6% (67.5–69.7)
Definition 5 Medical Claim Data	5686	4166	1520	73.3% (72.1–74.4)
Definition 6–1 Medical Claim Data	5025	3628	1397	72.2% (71.0–73.4)
Definition 6–2 Medical Claim Data	4323	3091	1232	71.5% (70.2–72.8)

With regard to the outcome definition for newly developed hyperthyroidism, the number of cases and PPV calculated using SS-MIX2 standardized storage data and medical claim data are shown in Table 7(*Number of cases and PPV according to the outcome definition for hyperthyroidism*). The PPV for hyperthyroidism, based on disease name definition alone, was 20–30%; however, the value exceeded 60% when the drug prescription was included in the conditions.

**Table 7:** Number of cases and PPV according to the outcome definition for hyperthyroidism.

Outcome Definition	Potential Case	True Case	Other Case	PPV
Data Source	A	B	C	B/A % (95%CI)
Definition 1				
SS-MIX2 Standardized Storage Data	1893	351	1542	18.5% (16.8–20.3)
Medical Claim Data	770	209	561	27.1% (24.0–30.3)
Definition 2				
SS-MIX2 Standardized Storage Data	722	442	280	61.2% (57.7–64.8)
Medical Claim Data	374	245	129	65.5% (60.7–70.3)
Definition 3–1				
SS-MIX2 Standardized Storage Data	603	402	201	66.7% (62.9–70.4)
Medical Claim Data	333	224	109	67.3% (62.2–72.3)
Definition 3–2				
SS-MIX2 Standardized Storage Data	447	313	134	70.0% (65.8–74.3)
Medical Claim Data	242	168	74	69.4% (63.6–75.2)
Definition 4 Medical Claim Data	653	197	456	30.2% (26.6–33.7)
Definition 5 Medical Claim Data	330	236	94	71.5% (66.6–76.4)
Definition 6–1 Medical Claim Data	318	221	97	69.5% (64.4–74.6)
Definition 6–2 Medical Claim Data	228	165	63	72.4% (66.6–78.2)

Method 2 was applied to two medical institutions and the subject outcome was defined as “newly developed acute renal failure.” A review of medical records was performed with the cooperation of specialists, including multiple nephrologists. For evaluation of the validity using gold standards regarding the information on medical records, the subjects accounting for 30% of the 1,447 potential cases were selected by random sampling. Based on the medical records, each case was classified as a “true case” or “other case.” Evaluation of definition 1 (disease name & acute elevation in serum Cre levels) based on medical record information showed that PPV was as low as 53.7% (Table 8: *Number of cases and PPV according to the outcome definition for acute renal failure*); however, this value increased to approximately 80–90% when patients who previously had high serum Cre levels were excluded.

**Table 8:** Number of cases and PPV according to the outcome definition for acute renal failure.

Outcome Definition 1)	Potential Case	Subjects	True Case	Other Case	PPV
		A	B	C	B/A % (95%CI)
Definition 1 Disease Name and Rapid Increase in Serum Creatinine Value					
All Data	1447	432	232	200	53.7% (49.0–58.4)
Previous serum Cre level ≤ 1.2 mg/dL	541	162	145	17	89.5% (84.8–94.2)
1.2 mg/dL≤ previous serum Cre level ≤ 2.0 mg/dL	807	241	194	47	80.5% (75.5–85.5)
2.0 mg/dL ≤ previous serum Cre level	640	191	38	153	19.9% (14.2–25.6)

1) Data Source was SS-MIX2 Standardized Storage Data.

## Discussion

This study investigated whether known adverse events could be detected by database surveillance using a hospital information system, in an attempt to substitute traditional large-scale surveys in which data from each hospital is individually collected by companies, etc. Study outcomes considered were newly developed diabetes, dyslipidemia, hyperthyroidism, and acute renal failure. The methods used in this study were relatively simple, because the review of laboratory examination results was performed by a single researcher in PMDA, although the review of medical records was done with the cooperation of multiple physicians. Detection of true cases by Method 2 took time and effort, as careful review of medical records by specialists was needed for validation. Contrastingly, Method 1 was more feasible and effective, detecting true cases and providing sufficient PPV only by reviewing the laboratory examination results; thus, this suggested the validity of the method. We believe that the simple validation performed using laboratory examination results alone is useful, if guideline definitions are specified.

In addition, a mini-sentinel surveillance showed a method for identifying true cases based on the information of test results alone and reported that the outcomes considered feasible for validation included hyperglycemia, dyslipidemia, hyperthyroidism, etc. [16].

The PPVs of hyperglycemia, dyslipidemia and hyperthyroidism were calculated only by laboratory examination results without reviewing medical records which required cooperation of specialists. This methodology was so effective to detect ADEs that large scale evaluation of ADEs was possible for many drugs which was used subsequent MID-NET project initiated in 2018 in Japan.

**Fig 3:**
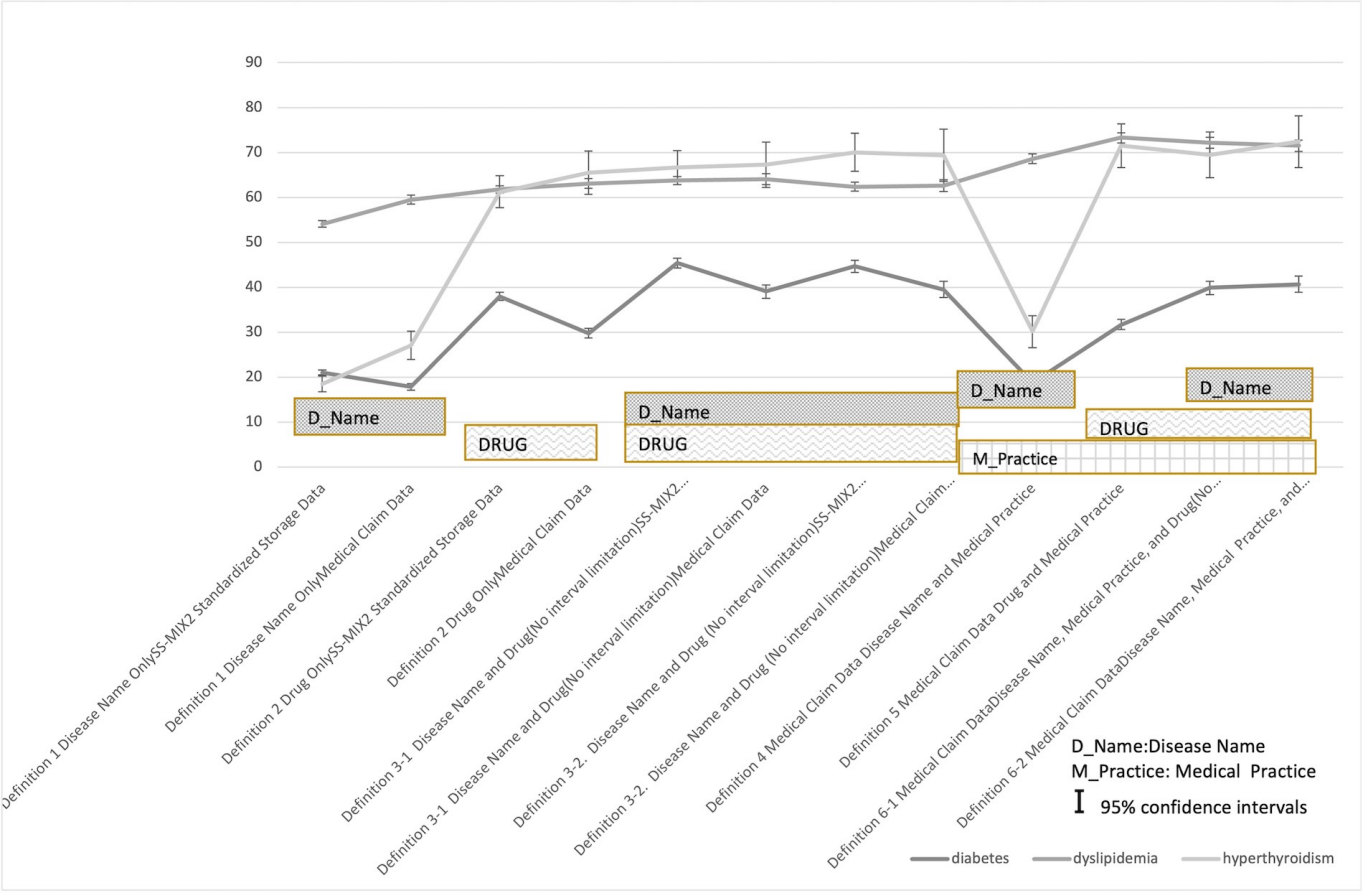
Positive Predictive Values (PPV)___Method 1.

SS-MIX Standard Storage Data was used for PPV definition. The reliability of SS-MIX Standardized Storage Data is generally accepted in Japan and also used in clinical studies such as diabetes mellitus [17]. We tried to graph each PPV about the definition of Method 1(Fig 3. Positive Predictive Values (PPV)___Method 1).There were fewer evaluation items and insufficient statistical analysis, but the following were shown.

The PPV for diabetes was relatively low. This may be attributed to the fact that evaluation was made based mainly on laboratory examination results, despite the inclusion of a wide variety of diabetes patients (those with suspected diagnosis, untreated patients, those under treatment, and those with different treatment effects) in the analysis. The PPV for diabetes was higher in the results obtained from SS-MIX2 standardized storage data than in those obtained from medical claim data in any of the definitions. The PPV for dyslipidemia was 50% or higher in all conditions. This may be due to the fact that many cases could be identified by the disease name definition alone, because of its high specificity. The PPV for hyperthyroidism outcome was 20–30% based on disease name definition alone; however, the value exceeded 60% when drug prescription was included in the definition, suggesting that drug prescription has high specificity for this disease. The total PPV for acute renal failure outcome was 53.7%, but increased to approximately 80–90% when patients who previously had high Cre levels were excluded. The PPV for this disease was higher than that of other diseases. Given that Cre levels are higher in patients on dialysis, etc., exclusion of such patients in this study might have led to higher PPV, but the most probable reason may be that “the definition was based on test results.” In addition, PPV based on disease name definition alone was low in all outcomes. The reason for this may be as follows: when medical institutions submit medical care fee claims, they are required to input disease names that are consistent with the medical practice provided; therefore, there may have been cases where the disease name recorded by the medical institution did not accurately reflect the actual clinical conditions. Thus, outcome definitions based on disease name alone may be inappropriate in studies using medical information databases.

This study shows that SS-MIX data extracted from multiple collaborating medical institutions can be used for quantitative study on safety assessment, such as the impact of safety measures, examination of adequacy of assessment and outcome definition. In the future, PMDA will promote the medical information database infrastructure development project in cooperation with the Ministry of Health, Labor and Welfare in order to build a medical information database for the purpose of improving safety measures for drugs, etc. [18].

In the system development of the medical information database, the same extraction program was sent to each medical institution based on the experience in the trial survey using the SS-MIX data so far, and the target data was executed by each medical institution. It has become possible to implement a system from the search and extraction of to the simple aggregation. Moreover, it is considered that the knowledge about the characteristics of the hospital information system data of medical institutions and the utilization method obtained from the trial survey using SS-MIX data such as this time can be utilized when using the medical information database in the future.

## Conclusion

When defining a disease, it is important to include the condition specific to the disease; furthermore, it is very useful if laboratory examination results are also included. Therefore, the inclusion of laboratory examination results in the definitions, as in the present study, was considered very useful for the analysis of multi-center SS-MIX2 standardized storage data. In Japan, it is expected that the number of pharmacoepidemiological studies based on the secondary use of large scale databases, such as medical information database, will increase in the future. Accordingly, the importance of validation studies is expected to increase as well. However, it is difficult to evaluate the validity of all outcome definitions using medical records. Therefore, the evaluation method of validity should be selected according to the types of outcomes. For example, the evaluation method using laboratory examination results can be used for diseases that can be diagnosed based on changes in laboratory values. Effective evaluation of the validity of the outcome definitions and accumulation of findings regarding various outcome definitions are considered important. In Japan, the 2018 MID-NET project, involving 24 hospitals (10 medical groups), was initiated based on the promising results presented here (i.e., direct detection of adverse events using EMR data) in an attempt to substitute post-marketing surveillance.
